# Autism in girls and the pre-referral environment

**DOI:** 10.1192/bjo.2021.740

**Published:** 2021-06-18

**Authors:** Peter Nussbaum

**Affiliations:** NHS Lothian

## Abstract

**Aims:**

This literature review sought to identify and highlight any sex specific factors in the diagnosis of autism spectrum conditions during the pre-referral period which might affect diagnosis rates in ASD in girls. The null hypothesis was that there are no sex specific factors that affect referral and diagnosis of ASD in girls.

**Background:**

Historically, boys are diagnosed with ASD more than girls but rates vary depending on clinical population characteristics. Diagnosis trends continue to demonstrate a large male excess. The concept of autism as a predominantly male condition has been challenged and there is increased focus on females with high functioning autism who are not being detected as easily.

Various theories exist as to why this is the case.

There are high rates of suicidality in ASD and risk of death by suicide is higher in ASD women (the reciprocal of the suicide rates in general population where more men complete suicide). Women with high functioning autism represent an at risk group. Undetected autism in females may be complicated by ‘camouflaging’ or masking of symptoms which puts a large strain on individuals functioning and mental health. Costs to society and the individual are large.

However, early identification and intervention improves outcomes such as activities of daily living and social behaviours.

**Method:**

An electronic literature search was completed using MEDLINE, PsycINFO and EMBASE in November 2018. Key terms were: (‘child*’ OR ‘adolescent’ OR ‘young pe*’) AND (‘ASD’ OR ‘autism’ OR ‘asperger*’ OR ‘high functioning*’ OR ‘PDD’ OR ‘Pervasive developmental*’) AND (‘girl*’ OR ‘sex’ OR ‘gender’). Papers were excluded on a number of grounds.

**Result:**

11 papers were included in the review from an initial 2823 abstracts.

**Conclusion:**

A number of papers highlighted important learning points. Some of the more original conclusions included that we require more studies comparing populations of girls with ASD to high risk, high functioning girls and female controls to clarify features particular to the ‘female phenotype’. Delays in diagnosis in girls appears to pre-date assessment so further thought on how to educate and support referral sources (caregivers and schools) on how to identify girls with autism is recommended and would be informed by further research focus on the previous point. Active case ascertainment should be considered in future research and follow-up of girls who do not receive a diagnosis at initial assessment were additional learning points that came from the review.

